# Amphipathic polymer-mediated uptake of trehalose for dimethyl sulfoxide-free human cell cryopreservation^☆^^☆☆^

**DOI:** 10.1016/j.cryobiol.2013.09.002

**Published:** 2013-12

**Authors:** Duncan M.C. Sharp, Andrew Picken, Timothy J. Morris, Christopher J. Hewitt, Karen Coopman, Nigel K.H. Slater

**Affiliations:** a Department of Chemical Engineering and Biotechnology, University of Cambridge, New Museum Site, Pembroke Street, Cambridge CB2 3RA, UK; b Centre for Biological Engineering, Department of Chemical Engineering, Loughborough University, LE11 3TU, UK

**Keywords:** Cryopreservation, Trehalose, Biopolymer, SAOS-2, PP-50

## Abstract

For stem cell therapy to become a routine reality, one of the major challenges to overcome is their storage and transportation. Currently this is achieved by cryopreserving cells utilising the cryoprotectant dimethyl sulfoxide (Me_2_SO). Me_2_SO is toxic to cells, leads to loss of cell functionality, and can produce severe side effects in patients. Potentially, cells could be frozen using the cryoprotectant trehalose if it could be delivered into the cells at a sufficient concentration. The novel amphipathic membrane permeabilising agent PP-50 has previously been shown to enhance trehalose uptake by erythrocytes, resulting in increased cryosurvival. Here, this work was extended to the nucleated human cell line SAOS-2. Using the optimum PP-50 concentration and media osmolarity, cell viability post-thaw was 60 ± 2%. In addition, the number of metabolically active cells 24 h post-thaw, normalised to that before freezing, was found to be between 103 ± 4% and 91 ± 5%. This was found to be comparable to cells frozen using Me_2_SO. Although reduced (by 22 ± 2%, *p* = 0.09), the doubling time was found not to be statistically different to the non-frozen control. This was in contrast to cells frozen using Me_2_SO, where the doubling time was significantly reduced (by 41 ± 4%, *p* = 0.004). PP-50 mediated trehalose delivery into cells could represent an alternative cryopreservation protocol, suitable for research and therapeutic applications.

## Introduction

In recent years, the developed world has seen an increase in demand for tissue replacement. While the number of donor organs and operations has remained relatively static, the number of patients on the transplant waiting list for kidney, pancreas, heart, lung, and liver has increased [31]. It is hoped that regenerative medicine, including stem cell-based therapies, could meet this need, as well as providing novel treatments for currently incurable conditions [39].

For stem cell-based therapies to be used routinely in a clinical setting, these cells must be stored and transported. Currently this need is met through cryopreservation, often using the cryoprotectant dimethyl sulfoxide (Me_2_SO). However, the viability of both adult and embryonic stem cells has been found to be significantly decreased by cryopreservation using Me_2_SO [20,42]. Perhaps more seriously, the functionality of cells can be adversely affected. For example, Katkov et al. [20] found that only 5–10% of human embryonic stem cells (hESCs) expressed the transcription factor Oct-4, a marker of pluripotency, following Me_2_SO cryopreservation. This property of facilitating the loss of hESCs pluripotency has been utilised in hESC differentiation protocols [14]. Cryopreservation using Me_2_SO may also have contributed to the failure of a phase III clinical trial using human mesenchymal stromal cells, due to loss of cell viability and functionality [17]. Indeed, it has been found that the genome-wide DNA methylation profiles of cells can be altered by Me_2_SO [40].

In addition, patients may experience severe side effects from Me_2_SO toxicity after cells preserved in this cryoprotectant are transplanted. These include cardiac arrest, severe respiratory arrest, severe neurotoxicity and epileptic seizures [12]. These side effects are thought to occur in one in 70 patients following autologous bone marrow transplantation [44]. Although this issue could be overcome by washing cells prior to implantation, this increases the complexity of the cell delivery method and could result in significant cell losses. Therefore there is a demand for Me_2_SO-free cryopreservation techniques, utilising non-toxic cryoprotectants, which maintain cell viability and functionality.

The non-permeating cryoprotectant trehalose may provide an alternative, however to provide maximum protection to the cells, the trehalose should be present on both sides of the cell membrane [15]. Recently, the amphipathic membrane permeabilising polymer PP-50 has been used to load human erythrocytes with trehalose, which led to a significant enhancement in cryosurvival [27]. PP-50, which can be removed from cell membranes by a small change in pH [26], is thought to be non-cytotoxic [11,22]. This is in stark contrast to previous studies using pore-forming bacterial toxins [1,6,15], where serious health concerns have been raised regarding the use of these proteins [32,41].

A number of alternative methods for the delivery of trehalose into cells have previously been employed [4,5]. These include the use of the ATP receptor channel P2X_7_ [7,8], prolonged cell culture [19] or endocytosis [18,30,33]. Stimulation of the P2X_7_ channel may lead to apoptosis, necrosis [2] or even neoplasia [3]. The latter two methods also have the disadvantage of requiring incubation times of 24 h or more. This is in contrast to the proposed method of PP-50 mediated trehalose delivery [27].

In the current study, the techniques for the cryopreservation of cells using trehalose and PP-50 developed by Lynch et al. [27] were extended to successfully preserve nucleated human cells. The Human osteosarcoma derived cell line SAOS-2 [16,35] was used as a model for nucleated, adherent human cells.

## Methods and materials

### Materials

Unless otherwise stated, all reagents were purchased from Sigma–Aldrich (UK). Materials for the PP-50 polymer synthesis were sourced as previously described [25]. Foetal bovine serum (FBS), l-glutamine, and penicillin/streptomycin were purchased from Invitrogen (UK). Dulbecco’s Phosphate-Buffered Saline (DPBS), 10 × DPBS and trypsin–EDTA were purchased from Life Technologies™ (UK). The CellTiter 96® AQueous One Solution Cell Proliferation Assay (MTS) was purchased from Promega (UK). The SAOS-2 cells were purchased from the European Collection of Cell Cultures. The Annexin V-FITC Apoptosis Detection Kit was purchased from BD Biosciences (UK).

### PP-50 synthesis and characterisation

The synthesis and characterisation of the PP-50 polymer were as previously described by Lynch et al. [25].

### Cell culture

SAOS-2 cells were grown in tissue culture flasks containing “growth media”: Dulbecco’s Modified Eagle’s Medium – high glucose (DMEM), supplemented with 10% (v/v) FBS, l-glutamine (2 mM), penicillin (100 IU/ml) and streptomycin (100 μg/ml). At approximately 70% confluency, the cells were subcultured with trypsin (0.05% w/v) and EDTA (0.02% w/v), and were subsequently split at a ratio of 1:6. The cells were maintained in a humidified incubator at 37 °C with 5% CO_2_. The cells were used between passages 4 and 20.

### Calcein and propidium iodide fluorescence assay

Calcein, which is membrane impermeable, was used as a tracer for hydrophilic species delivery into the cells. The viability of the cells was assessed using propidium iodide (PI) staining.

SAOS-2 cells were seeded into 35 mm glass bottom culture dishes (PAA, UK) at 2 × 10^5^ cells/dish, in growth media. After 48 h of incubation in a humidified incubator at 37 °C with 5% CO_2_, a positive control for PI staining was prepared by fixation with paraformaldehyde solution (4% w/v, in DPBS) for 10 min, followed by washing (×3) with DPBS.

For the remaining dishes, the cells were washed twice with DPBS. Afterwards, the cells were incubated for 4 h in serum-free media supplemented with 0.2 M trehalose, 2 mM calcein, and with or without PP-50 (200 μg/ml), at pH 7.05. The cells were washed twice with DPBS, and incubated with growth media containing Hoechst 33342 (2 μg/ml) and PI (2 μg/ml) for 15 min. Following three washes with DPBS, the cells were imaged using a TCS SP5 inverted laser scanning confocal microscope (Leica, Germany).

### PP-50 toxicity

SAOS-2 cells were seeded into 96-well tissue culture plastic plates (Corning, UK) at 5000 cells/well. After 24 h, the cells were washed twice with DPBS at either pH 7.4 or pH 7.05. The cells were incubated (37 °C with 5% CO_2_) in serum-free growth media containing different PP-50 concentrations (0–1000 μg/ml) at pH 7.4 or pH 7.05, for 2 or 24 h. The cells incubated for 2 h were subsequently washed with DPBS and incubated for 22 h in growth media. An MTS assay was performed at the 24 h mark according to the manufacturer’s instructions.

### Cryopreservation and reconstitution

SAOS-2 cells were seeded into 6-well tissue culture plastic plates (Corning, UK) at 10^5^ cells/well. After 24 h, the cells were washed with DPBS (pH 7.4), then DPBS (pH 7.05), and were then incubated (37 °C with 5% CO_2_) in “incubation media”: serum-free media with 0.2 M trehalose, with or without PP-50 at different concentrations, and water (18.2 MΩ.cm, Milli-Q® filtered, Millipore, USA), at pH 7.05. Following incubation, the osmolarity of all solutions was adjusted to that of the incubation media using 10 × DPBS (PAA, UK) and/or water unless otherwise stated.

After 2 h of incubation (37 °C with 5% CO_2_), the cells were washed twice with DPBS, and trypsin/EDTA was added at 200 μl/well. After 15 min of incubation (37 °C with 5% CO_2_), 500 μl/well growth media was added and the cells were centrifuged at 350*g* for 5 min and resuspended in 150 μl of 0.2 M trehalose in FBS. Controls using un-incubated cells were also prepared and resuspended in FBS (90%) and Me_2_SO (10%). All samples were transferred into cryovials (Greiner, UK), and transferred into an isopropanol freezing container (Nalgene, USA), then passively cooled in a −80 °C freezer overnight, before storage in vapour-phase liquid nitrogen for at least 48 h.

The cells were subsequently thawed by immersing the cryovials in a 37 °C water-bath, after which 850 μl/cryovial of growth media were slowly added. After centrifugation, the cells were resuspended in growth media, and added to the wells of 96-well plates (100 μl/well). Non-frozen SAOS-2 cells were seeded into the plates at 5000 cells/well. After 4 h of incubation (37 °C with 5% CO_2_), the media was changed to growth media of normal osmolarity. MTS assays were subsequently performed at 24, 48 and 72 h, according to the manufacturer’s instructions.

(1) $$t_{d}=(t_{2}-t_{1})\frac{\ln2}{\ln\frac{N(t_{2})}{N(t_{1})}}$$

(2) $$N(0)=\frac{N(24)}{2^{\frac{24}{t_{d}}}}$$

The number of metabolically active cells was found using a standard curve. The doubling times, *t_d_*, were calculated using Eq. (1), where *t*_1_ and *t*_2_ represent the time at time-points 1 and 2, respectively, and *N*(*t*_1_) and *N*(*t*_2_) represent the number of cells at time-points 1 and 2, respectively. The numbers of proliferative cells immediately post-thaw were estimated using Eq. (2).

### Viability and apoptosis assay

SAOS-2 cells were seeded into 25 cm^2^ tissue culture flasks (Corning, UK) at 5 × 10^5^ cells/flask. After 24 h, the cells were frozen as described above, scaling volumes appropriately for the growth area. The freezing protocols used were; 0.2 M trehalose (additional 133 mOsm/l) with or without 25 μg/ml PP-50, and the Me_2_SO control. Following thawing of the cells, using a 37 °C water-bath, an Annexin V/PI flow cytometry assay was performed. The manufacturer’s instructions were followed, with the modification that for the cells frozen with trehalose, the osmolarity of all reagents was adjusted with an additional 133 mOsm/l of sodium chloride. The samples were examined using a FACScan flow cytometer (Becton Dickinson, USA).

### Statistical analysis

All statistical data analysis was performed using the statistical software package SPSS 14.0 for Windows. The data for the numbers of metabolically active cells at 24 h post-thaw, the doubling times and the flow cytometry data were analysed by one-way ANOVA followed by Tukey HSD. Values of *p* < 0.05 were considered to be statistically significant [45].

All data quoted represent the mean of three repeats ± the standard error of the mean (SEM), unless otherwise stated.

## Results

### Calcein and propidium iodide fluorescence assay

**Fig. 1:**
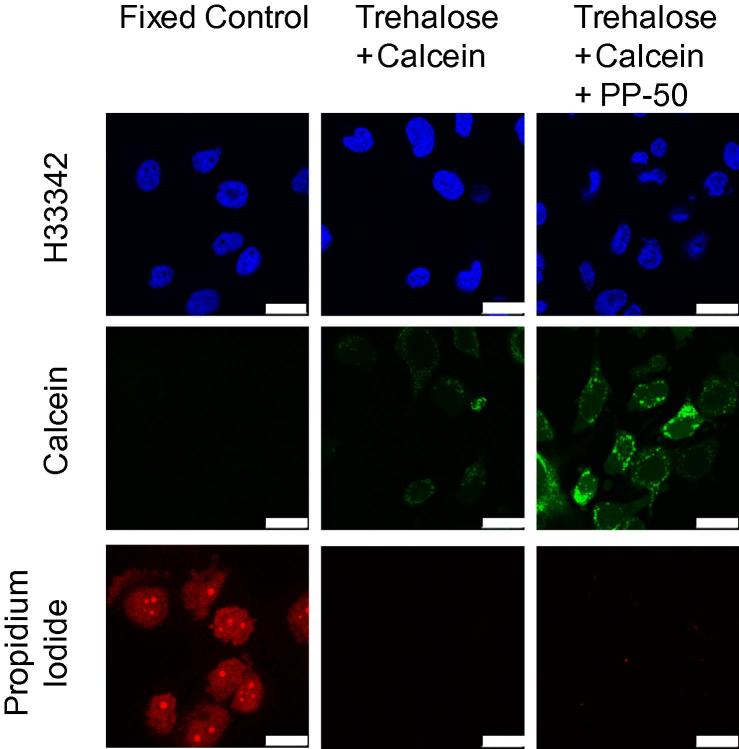
Representative fluorescence microscopy images of SAOS-2 cells either fixed with 4% PFA or incubated with 0.2 M trehalose, 2 mM calcein, with or without PP-50 (200 μg/ml), at pH 7.05. The cells were subsequently stained with Hoechst 33342 (2 μg/ml) and PI (2 μg/ml) for 15 min, following three washes with DPBS. The scale bars represent 25 μm.

Cells incubated in the presence of trehalose and calcein stained weakly with calcein (Fig. 1). The calcein staining of the cells in the presence of the cell permeabilising polymer PP-50 was found to be stronger. For the non-fixed cells, no PI positive cells were observed.

### PP-50 toxicity

**Fig. 2:**
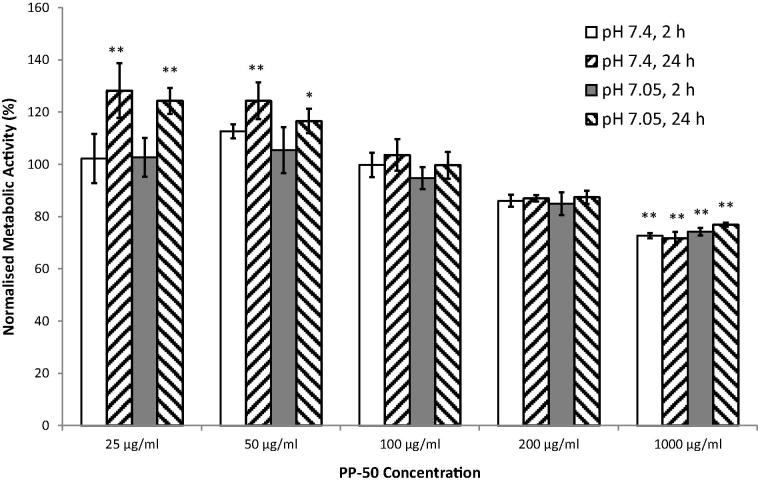
Metabolic activity of SAOS-2 cells 24 h after exposure to PP-50 at difference concentrations for either 2 or 24 h. These data were normalised to cells incubated in the absence of PP-50, at the same pH and incubation time, and were derived from three replicates. * and ** denote significant differences of *p* < 0.05 and *p* < 0.01, respectively from the no PP-50 controls.

In the experimental range tested, it was found that pH had no significant effect on metabolic activity (Fig. 2). PP-50 at 1000 μg/ml significantly decreased metabolic activity for all incubation conditions tested. For PP-50 concentrations ⩽50 μg/ml, there was a small but statistically significant increase in metabolic activity when the cells were incubated for 24 h in the presence of the polymer.

### Cryopreservation and reconstitution

**Fig. 3:**
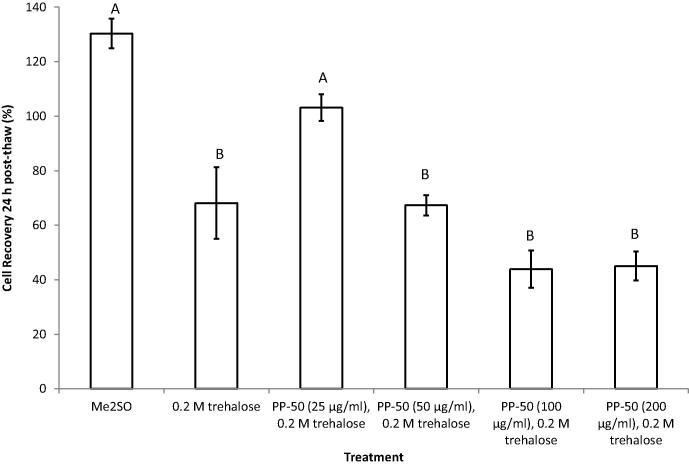
Relationships between cryopreservation treatment and the number of metabolically active cells present 24 h after thawing, normalised by the pre-freeze cell numbers. These data were derived from three replicates. Statistical differences of the means (*p* < 0.05) are denoted where the letters differ from one another.

The number of metabolically active cells present 24 h post-thaw, was determined from the MTS assay. These data were normalised by the number of cells present in the pre-freeze samples, taking dilution into account (Fig. 3). The post-thaw recovery of the cells incubated with trehalose in the absence of PP-50 was found to be 68 ± 5%. Of the concentrations tested, only 25 μg/ml of PP-50 in the pre-freeze incubation media was found to significantly enhance the cell recovery (103 ± 4%, *p* = 0.034). Although the cell recovery was greater in the Me_2_SO control group (130 ± 14%), this was found not to be statistically significant. The fact that this group had a higher 24 h post-thaw recovery than 100%, may be explained by proliferation of the cells during the first 24 h. Making the assumption that the different cell doubling times, specific to each treatment group, remained the same throughout the experiment, the number of viable cells capable of proliferating immediately post-thaw was calculated to be 64 ± 5% and 70 ± 11% for the PP-50/trehalose and Me_2_SO treatments, respectively. Using the same calculation, the number of proliferative cells for the non-frozen control was 116 ± 6%.

**Fig. 4:**
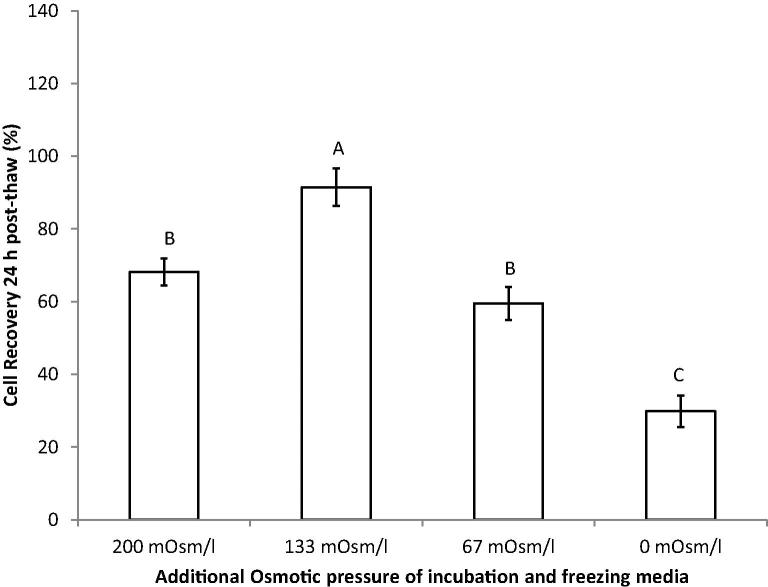
Relationships between the additional osmotic pressure and the number of metabolically active cells present 24 h after thawing, normalised by the pre-freeze cell numbers. These data were derived from three replicates. Statistical differences of the means (*p* < 0.05) are denoted where the letters differ from one another.

For the freezing protocol involving PP-50 and trehalose, the osmolarity of the incubation and freezing media was optimised (Fig. 4). The optimum additional osmolarity was found to be 133 mOsm/l, with a 24 h cell recovery of 91 ± 5%.

**Fig. 5:**
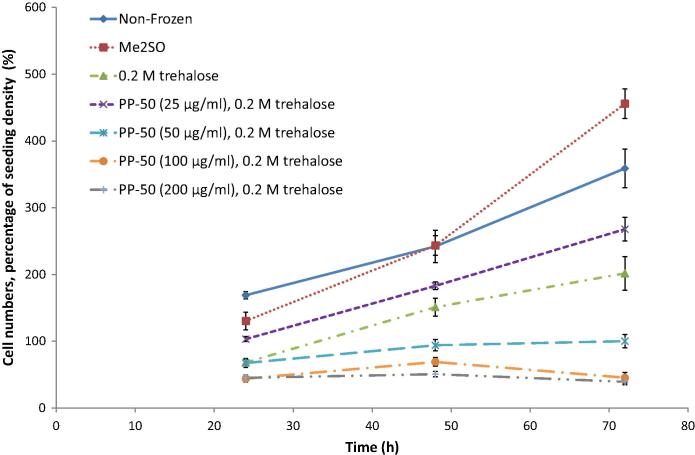
Relationships between cryopreservation treatment and the number of metabolically active cells present up to 72 h post-thaw, normalised by the pre-freeze cell numbers. These data were derived from three replicates.

**Fig. 6:**
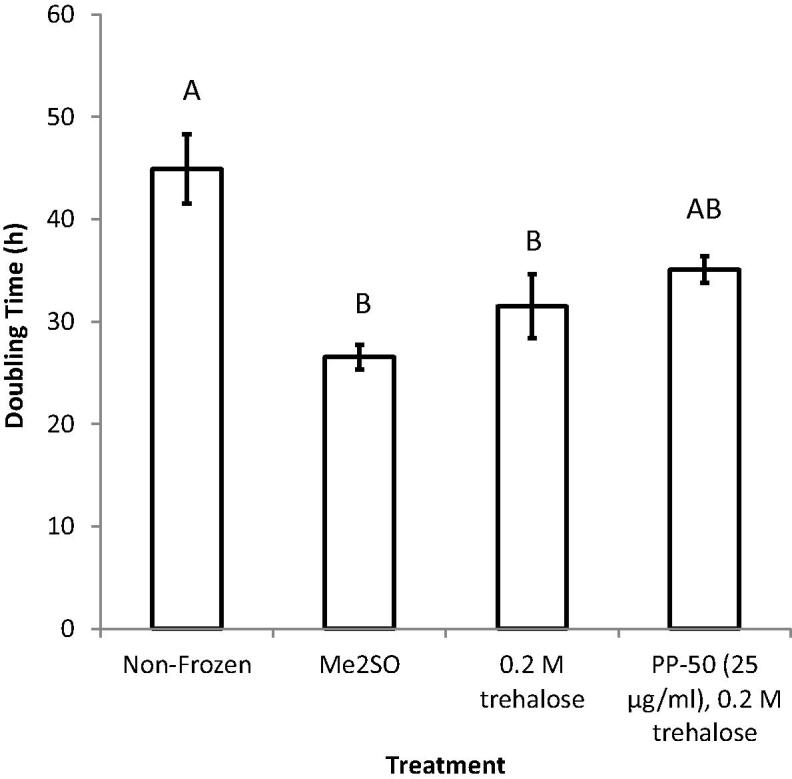
Relationships between the doubling times for metabolically active SAOS-2 cells, measured between 24 and 72 h post-thaw. These data were derived from three replicates for each time-point. Statistical differences of the means (*p* < 0.05) are denoted where the letters differ from one another.

The proliferation of the SAOS-2 cells post-thaw was examined (Fig. 5). It was found that when the concentration of PP-50 in the incubation media was ⩾50 μg/ml, the cells did not proliferate at the normal rate compared to cells kept in continuous culture (non-frozen control). However, when the concentration was ⩽25 μg/ml the growth curves were similar to the non-frozen control. This was also reflected in the doubling times for the cells. Although reduced (by 22 ± 2%, *p* = 0.09) these two groups were not significantly different from the non-frozen control (Fig. 6).

In contrast, the cells frozen using Me_2_SO were found to have an abnormally high rate of growth. This was also reflected in the doubling time for the cells (Fig. 6), which for this group was significantly different from the non-frozen control during the test period (reduced by 41 ± 4%, *p* = 0.004).

### Viability and apoptosis assay

**Fig. 7:**
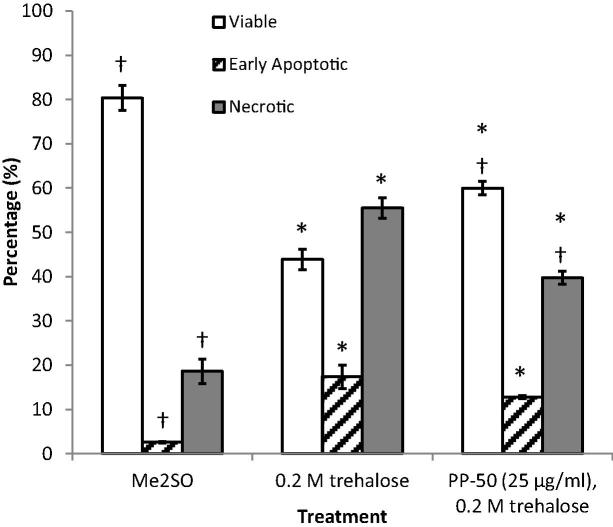
The viability of the SAOS-2 cells was assessed immediately post-thaw using an Annexin V-FITC/PI kit by flow cytometry. Cells negative for both Annexin V and PI are considered as viable. Cells positive for Annexin V, but not PI were scored as early apoptotic. These data were derived from three replicates. Cells positive for PI were considered to be necrotic. * and † denote significant differences of *p* < 0.05 from the Me_2_SO and the 0.2 M trehalose controls, respectively.

To determine the cell cryosurvival, the post-thaw viability of the cells was determined by flow cytometry using Annexin V-FITC and PI staining (Fig. 7). The percentage of viable cells was significantly higher for the cells frozen using Me_2_SO (80 ± 3%) than for either treatment using trehalose with or without PP-50 (60 ± 2%, and 44 ± 3%, respectively). The addition of PP-50 at 25 μg/ml during the incubation step, significantly enhanced viability (by a factor of 37 ± 7%, *p* = 0.002). For all the treatment groups tested, the majority of the non-viable cells were found to be necrotic rather than apoptotic.

## Discussion

Perhaps the two most important criteria with which different methods of cell cryopreservation should be judged are; cryosurvival and retention of normal cell processes. The latter is thought to be particularly important for both research and therapeutic applications. Here, a Me_2_SO-free cryopreservation protocol, using trehalose delivery utilising PP-50, was developed and assessed. The cell line SAOS-2 was used as a model for nucleated, adherent human cells.

### Calcein and propidium iodide fluorescence assay and PP-50 toxicity

Calcein, like trehalose, is thought to be impermeable to the cell membrane. Calcein has therefore been used in previous studies to assess the extent of delivery of hydrophilic species into cells [10,11]. The degree of calcein uptake in the presence of the PP-50 was less than that previously reported for the related polymer PP-75 [10,11]. In part, this may be explained by the presence of trehalose in the incubation media in the studies described above. This increase in osmotic pressure caused by the trehalose supplementation of the media, may have decreased the rate of endocytosis for the cells [34]. Endocytosis has previously been found to play an important role in the delivery of hydrophilic species into cells using the related polymer PP-75 [21]. However since the delivery of trehalose into human erythrocytes which do not perform endocytosis, has previously been demonstrated [27], delivery through the cell membrane may also be important. It was concluded that PP-50 was capable of delivering hydrophilic species, such as trehalose, into cells. It should be noted that the PP-50 appeared to increase the rate of uptake of hydrophilic species by endocytosis compared to the control (Fig. 1). In addition to the release of trehalose into the cytoplasm, trehalose in endosomes may also have contributed to the efficacy of the PP-50/trehalose cryopreservation protocol [33].

Propidium iodide which is incapable of staining cells with intact cell membranes, has been widely used to assess the viability of cells [11,28,38]. In the experiments described above, PI staining was used to determine the viability of the cells, and whether the membrane permeabilising effect of the PP-50 could be reversed by washing with pH 7.4 DPBS. Previous studies have found that the hydrophobicity of PP-50 is strongly affected by pH. The polymer’s ability to bind to the hydrophobic core of cell membranes is thought to be significantly higher at pH 7.05 than at pH 7.4 [25]. Indeed, this pH change has been found to be sufficient to remove PP-50 bound to cell membranes [26]. For the group previously permeabilised by PP-50, no PI positive cells were observed (Fig. 1). These data suggest that the permeabilising effect of PP-50 is reversible and is in agreement with previous studies by Lynch et al. [26].

The metabolic activity of SAOS-2 cells was assessed after either a 2 or 24 h challenge with PP-50. This was conducted both at pH 7.05, at which the polymer is thought to have a permeabilising effect on cell membranes, and pH 7.4, at which the polymer is thought not to associate with cell membranes. No toxic effect was observed for PP-50 concentrations ⩽200 μg/ml. No significant decrease in metabolic activity was observed for these polymer concentrations at both permeabilising and non-permeabilising pHs (Fig. 2). In addition, no PI positive cells were observed when incubated with PP-50 at 200 μg/ml (Fig. 1). This was in agreement with previous studies [11,22].

Interestingly, there was a small but statistically significant increase in metabolic activity when the cells were incubated for 24 h in the presence of the polymer. This may be due to the cells under “serum starving” conditions, metabolising the PP-50. Alternatively, the cells may have been more metabolically active in response to loss of elements from the cytoplasm, caused by membrane permeabilisation by the PP-50.

### Cryopreservation and reconstitution

Extracellular concentrations of 0.2 M trehalose have previously been used in the cryopreservation of nucleated mammalian cells [6,9,15,29]. Since the osmotic coefficient of trehalose in aqueous solutions is 1.01 [43], 0.2 M trehalose yields an increase in osmolarity of approximately 200 mOsm/l. Increasing the normal osmolarity of media by more than 200 mOsm/l, can lead to apoptosis of the majority of cells [13]. Lynch et al. [27] had found that altering the PP-50 concentration in the presence of trehalose in the incubation media, determined the resulting intracellular trehalose loading. The concentration of PP-50 in the incubation media was therefore altered to determine the polymer concentration leading to an optimal delivery of trehalose into the cells. Of the concentrations tested, the optimum PP-50 concentration was found to be 25 μg/ml. This was both in terms of the cell recovery at 24 h post-thaw, and minimising differences in doubling time from the non-frozen control.

Freezing media consisting of 10% Me_2_SO and 90% FBS was chosen as the control cryopreservation media. Media such as this has been widely used in previous studies [23,36,37]. The 24 h cell recovery for the optimum PP-50 concentration (103 ± 4%) was found to be less than that for the Me_2_SO control (130 ± 14%), although this difference was not statistically significant. In part, this may be explained by proliferation of the SAOS-2 cells during the first 24 h post-thaw. Assuming the cell doubling times remained constant throughout the experiment, the number of viable cells capable of proliferating immediately post-thaw for the PP-50/trehalose and Me_2_SO protocols was estimated to be comparable (64 ± 5% and 70 ± 11%, respectively). This estimated cryosurvival was similar to that achieved for mesenchymal stem cells by Wang et al. [42]. Hence the cryosurvival of proliferative cells achieved using the PP-50/trehalose treatment may have been comparable to the Me_2_SO control. It should be noted that MTS assays were not performed on the cells immediately post-thaw, as the presence of early apoptotic cells can yield misleading results [24], as could the presence of cells incapable of substrate attachment.

The cryosurvival immediately post-thaw was tested further for these protocols, using a flow cytometry based Annexin V/PI assay. The proportion of viable cells for the PP-50/trehalose and Me_2_SO protocols were found to be comparable to those calculated above (80 ± 3% and 60 ± 2%, respectively). This could indicate that there is not a significant sub-population of cells for either protocol that appears viable, but is non-proliferative during subsequent culture.

As discussed previously, Me_2_SO is currently the cryoprotectant of choice for most cell culture and therapeutic applications. Although there is scope for improving the number of cells that survive the freezing process, the two most concerning problems associated with the use of Me_2_SO are loss of cell functionality, and toxicity to patients. Therefore, of the outcome measures tested, the comparison of the cell doubling times to the non-frozen control was thought to be the more important. It was found that the rate of proliferation was abnormally high for the cells cryopreserved using Me_2_SO compared to non-frozen SAOS-2 cells (Fig. 5). Indeed the cell doubling times were found to be significantly different from the non-frozen control by 41 ± 4%. In contrast, the doubling time for the cells cryopreserved using the optimum PP-50/trehalose protocol did not significantly affect the doubling time (Fig. 6).

These data suggest that the normal processes of the cells were affected less when cryopreserved using PP-50/trehalose than Me_2_SO, while maintaining high cell recovery. In addition, cells cryopreserved using this technique, would not contain traces of Me_2_SO, and therefore have the potential to reduce the side effects experienced by patients receiving transplants of cryopreserved cells. Subject to future development and testing, PP-50 mediated delivery of trehalose into cells could represent an alternative to conventional cell cryopreservation protocols for both therapeutic and research applications.

## Conclusions

In this study, the feasibility of a cellular cryopreservation protocol, utilising PP-50 mediated delivery of trehalose into cells, was assessed using SAOS-2 cells. The concentrations of PP-50, as well as the osmotic pressure of the incubation and freezing solutions, were optimised. The optimum PP-50/trehalose cryopreservation protocol yielded comparable cell recovery at 24 h post-thaw to cells cryopreserved using Me_2_SO. Cryopreservation using the PP-50/trehalose protocol, did not significantly affect the cell doubling time, in contrast to Me_2_SO cryopreservation. After future development and testing, delivery of trehalose utilising PP-50, could form the basis of a cryopreservation protocol superior and safer to those based on Me_2_SO, for research and therapeutic applications.
